# Global ecological niche conservatism and evolution in *Olea* species

**DOI:** 10.1016/j.sjbs.2022.103500

**Published:** 2022-11-11

**Authors:** Uzma Ashraf, A. Townsend Peterson, Muhammad Nawaz Chaudhry, Marlon E. Cobos

**Affiliations:** a Department of Land, Air and Water Resources, University of California, Davis, USA; b Wild Energy Initiative, Institute of the Environment, University of California, Davis, USA; c Biodiversity Institute, University of Kansas, Lawrence, KS, USA; d Department of Environmental Sciences and Policy, Lahore School of Economics, Lahore, Punjab, Pakistan

**Keywords:** Ancestral reconstruction, Climate change, Evolution, Niche evolution in olives, Phylogeny, Oleaceae, Olives

## Abstract

**Background and Aims:**

Climate is an important parameter in delimiting coarse-grained aspects of fundamental ecological niches of species; evolution of these niches has been considered a key component in biological diversification. We assessed phylogenetic niche conservatism and evolution in 24 species of the family Oleaceae in relation to temperature and precipitation variables. We studied niches of 17 *Olea* species and 7 species from other genera of Oleaceae globally.

**Methods:**

We used nuclear ribosomal and plastid DNA to reconstruct an evolutionary tree for the family. We used an approach designed specifically to incorporate uncertainty and incomplete knowledge of species’ ecological niche limits. We performed parsimony- and likelihood-based reconstructions of ancestral states on two independent phylogenetic hypotheses for the family. After detailed analysis, species’ niches were classified into warm and cold niches, wet and dry niches, and broad and narrow niches.

**Key Results:**

Given that full estimates of fundamental niches are difficult, we explore the alternative approach of explicit incorporation of knowledge of gaps in the information available, which allows avoidance of overestimation of amounts of evolutionary change. The result is a first synthetic view of evolutionary dynamics of ecological niches and distributional potential in a widespread plant family. Temperate regions of the Earth were occupied only by lineages that could derive with cold and dry niches; Southeast Asia held species with warm and wet niches; and parts of Africa held only species with dry niches.

**Conclusions:**

High temperature in Lutetian (Oligocene) and low temperature in Rupelian (Eocene) with major desertification events play important role for niche retraction and expansion in the history for Oleaceae clades. Associations between environmental niche characteristics and phylogeny reconstruction play an important role in understanding ecological niche conservatism, the overall picture was relatively slow or conservative niche evolution in this group.

## 1 Introduction

Climate change is a major factor in shaping geographic distributions of plant species (Thuiller et al., 2005), and (in the longer term) a significant selective force that causes evolutionary change (Franks et al., 2014). Ecological niches are features of species and populations that summarize population-level responses to climate (and other dimensions of the environment), and as such are the target of natural selection (Lewontin, 1970, Walther et al., 2002) and biotic interactions (Ashraf et al., 2021). Nonetheless, the degree to which niches are conserved or are dynamic in evolving lineages remains a matter of debate (Losos, 2008, Araújo et al., 2013). Several historical and ecological factors may increase or decrease the likelihood of niche change in evolving lineages, but these factors and their relative roles remain poorly known. The potential for evolution of climatic niches of species is thus important for understanding past and future climate impacts on biodiversity (Ribeiro et al., 2017), with diverse implications in evolution and ecology (Warren et al., 2008).

The concept of ecological niche conservatism was first analyzed explicitly on geographic scales by Peterson et al. (1999). Numerous methods have since been used to address these questions, including ecological niche models (Evans et al., 2009), niche overlap metrics (Warren et al., 2008, Petitpierre et al., 2012), phyloclimatic analysis (Ribeiro et al., 2017), and other approaches, although in many cases inappropriate choices of methods have led to incorrect conclusions (see analyses in Peterson, 2011, Saupe et al., 2017). Phyloclimatic analysis is a combined analysis of phylogenetic patterns and environmental distributions of species (Evans et al., 2009, Ahmadzadeh et al., 2013, Ribeiro et al., 2017): niches are analyzed in the context of the phylogeny of a lineage, and patterns of stability or change are assessed. Such analyses give rates of niche change and shifts of geographic distributional potential of species in the group under study. However, ecological niches and phylogenetic niche conservatism (PNC) are defined differently by different authors, which has led to misunderstandings and debate (Kozak and Wiens, 2010, Peterson, 2011, Saupe et al., 2017). Specifically, in PNC analysis, a key factor is that of considering the full set of abiotic conditions that make up the fundamental niche (Peterson, 2011), yet environmental conditions across present-day distributions often limit the ability to view and assess fundamental ecological niches (Saupe et al., 2017). Failure to consider these limitations leads to overestimation of evolutionary plasticity of niches (Saupe et al., 2017).

**Fig. 1:**
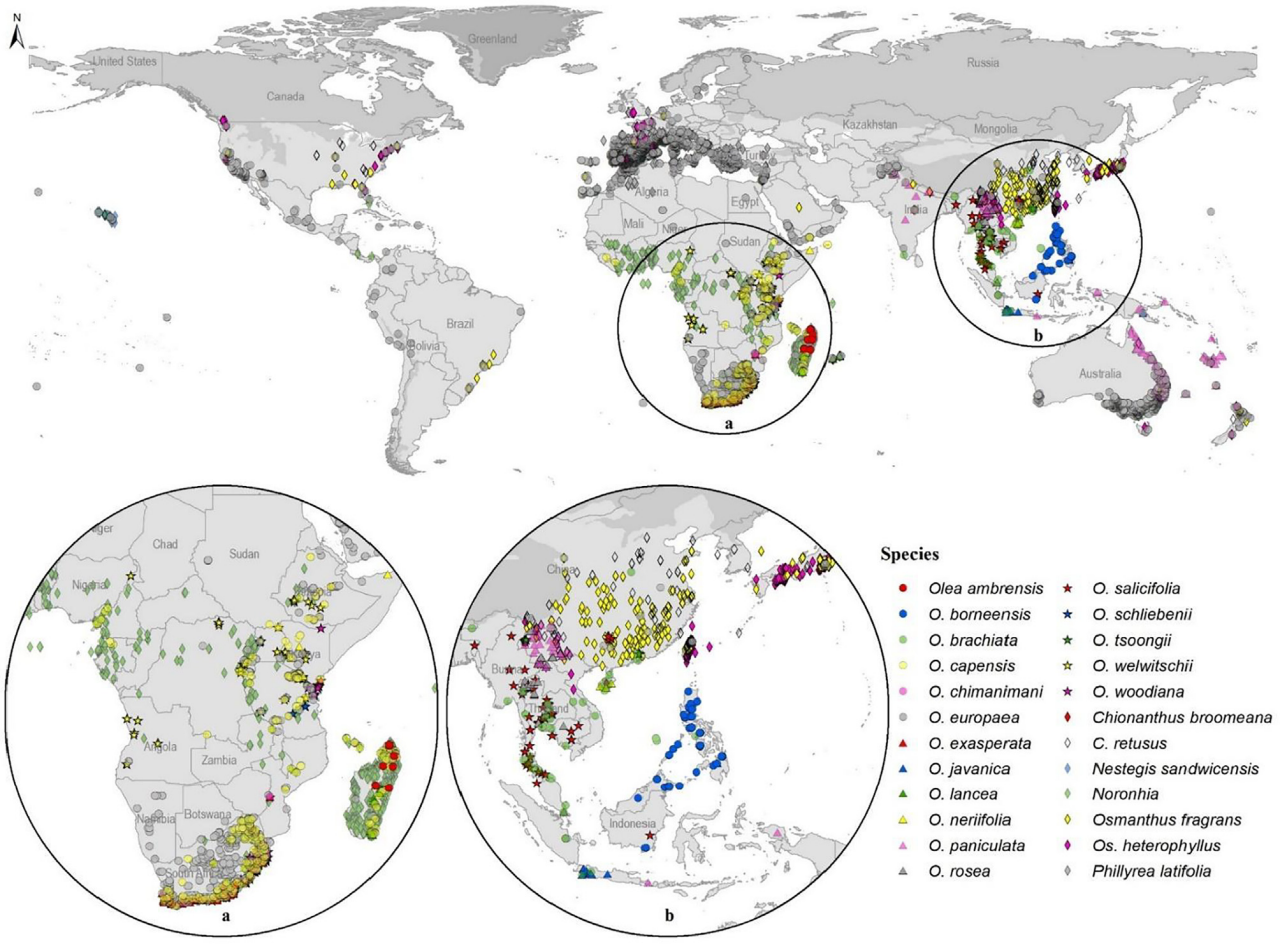
Global summary of occurrences of species of six genera of Oleaceae.

Here, we focus on the olives and related species of Oleaceae (Besnard et al., 2009). The family includes 25 genera, one of which is extinct (Wallander & Albert, 2000). *Olea europaea* is distributed worldwide, with introduced populations in New Zealand, Australia, and the Pacific islands, thanks to human-mediated dispersal (Besnard et al., 2007, Ashraf et al., 2017). Other Oleaceae species have smaller distributions, but species of the family are distributed across Africa, Asia, and Europe (Fig. 1; Besnard et al., 2009). Previously, this group was explored via biogeographic (Ashraf et al., 2016, 2017 & 2021) and phylogenetic (Besnard et al., 2001, 2007, 2009, 2011 & 2013) perspectives separately, but these two dimensions with environmental factors have never been explored in tandem, to assess niche conservatism in the group. Here, we explore and assess the traits of the *Olea* species and how they have been conserved over the evolutionary history of evolution in this group.

This study aims to evaluate and reconstruct evolutionary changes in fundamental ecological niches in the Oleaceae. We reconstructed ancestral states of niches related to temperature and humidity for species in the family based on climatic characteristics of known occurrences of the various species. An important point is that we incorporated explicit hypotheses of access to conditions, which avoids rampant overestimation of evolutionary niche dynamics (Owens et al., 2020, Saupe et al., 2017). We studied niches of 17 *Olea* species and 7 species from other genera of Oleaceae. We related these niche estimates to two independent phylogenetic hypotheses based on analysis of five gene regions (Besnard et al., 2009). The result is a first synthetic view of evolutionary dynamics of ecological niches and distributional potential in a widespread plant family.

## 2 Methods

**Fig. 2:**
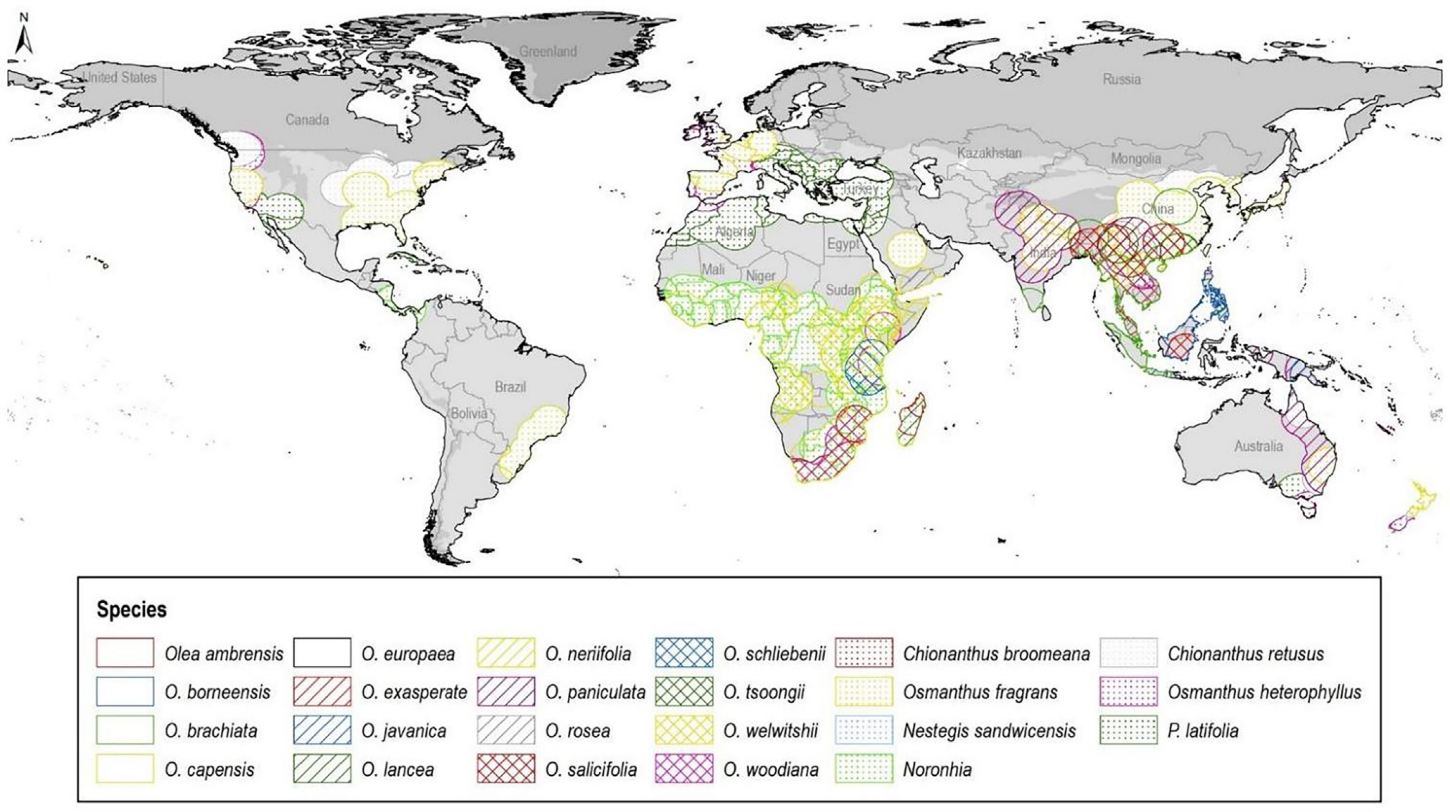
Accessible area (**M**) hypotheses for each species of Oleaceae in phylogenetic analyses based on 500 km buffers around known occurrences.

This analysis was based on profiles of use and availability of climatic conditions across the geographic ranges of individual species of the family Oleaceae. We considered 24 species, reflecting the set of species included in the most complete phylogenetic analysis of the family to date (Besnard et al., 2009). Since *Olea europaea* is distributed nearly globally, whereas other species have more restricted distributions (Fig. 1), we began with a global terrestrial extent (excluding Antarctica) for our analyses (Fig. 2). However, we defined more restricted areas of analysis for each species, based on 500 km buffers around known occurrences that were later reduced to remove areas likely not accessible to the species (e.g., removing Sumatra for a Javan endemic species). Individual areas were thus restricted as follows: Madagascar for *Olea ambrensis* and *O. lancea*; Malaysia and Philippines for *O. borneensis*; parts of Southeast Asia for *O. brachiata, O. rosea, O. salicifolia, Chionanthus broomeana, Osmanthus heterophyllus, O. fragrans,* and *Chionanthus retusus*; all or part of Africa for *Olea capensis, O. exasperata, O. neriifolia, O. schliebenii, Noronhia, O. woodiana,* and *O. welwitschii*; Mediterranean region for *P. latifolia*; Papua New Guinea, Philippines, and Indonesia for *O. javanica*; Mozambique for *O. chimanimani*; and Australia and South Asia for *O. paniculata*. See Fig. 1 for a map of these analysis areas.

We downloaded primary occurrence data for each of the species from the Global Biodiversity Information Facility (GBIF; https://www.gbif.org/), *species*Link (https://splink.cria.org.br/), iDigBio (https://www.idigbio.org), and REBIOMA (https://data.rebioma.net/). Although the family comprises 25 genera and ∼ 600 species (Wallander and Albert, 2000, Besnard et al., 2009), we focused on these 24 species taxa, combining intraspecific taxa and multiple individuals within species that were included in the phylogenetic analysis. Species represented in the phylogenetic trees by multiple individuals with different placements or branch lengths were reduced to single representatives by randomly selecting a single individual; these random choices were repeated 10 times to minimize error. Results of the 10 replicate analyses were combined for final results. Occurrence data were analyzed critically to remove points falling outside the species’ known native ranges (e.g., in botanical gardens), as determined by consultation of the literature and expert opinion (Contento et al., 2002, Rugini et al., 2011). Records with textual locality descriptors but lacking geographic coordinates for species for which data were otherwise few (*O. rosea, O. javanica, O. lancea, O. exasperata,* and *O. chimanimani*) were georeferenced by consulting to Google Earth (Patterson, 2007).

For ancestral state reconstructions of environmental characters, we used two alternative phylogenetic trees both from Besnard et al. (2009). That study used nuclear ribosomal and plastid DNA to reconstruct an evolutionary tree for the family; the final trees from that study were kindly provided by the authors of the study. Tree 1 was based on four plastid DNA regions (*trnT-trnL, trnL-trnF, trnS-try* and *matK*). Tree 2 was based on nuclear ribosomal DNA (ITS-1). Phylogenetic tree and environmental range datasets were harmonized via careful checking of species’ nomenclature and geographic ranges, and species not represented in both datasets were removed (see topologies of two trees, in Fig. 5s). Certainly, one shortcoming of our analysis is that we were able to assess only ∼ 3.5 % of the species in the family and 50.5 % of species in the genus *Olea*, as only these species were included in the phylogenetic analysis. We received time-calibrated ultrametric trees from Besnard et al. (2009), which were used as is in analyses in Mesquite (version 2.1; Maddison & Maddison, 2007). For further analysis, we calibrated ultrametric rooted trees using the phytools R package (R Core Team, 2021, Revell, 2012) for niche evolution analysis in R.

To add environmental information relevant to the Oleaceae, MERRAclim climate data layers summarizing temperature and specific humidity were downloaded from https://datadryad.org//resource/https://doi.org/10.5061/dryad.s2v81, including temperature and specific humidity data for 2000–2010, at 2.5′ spatial resolution (Vega et al., 2017). We chose 2000–2010 specifically to coincide with the modal decade among the occurrence data. These datasets comprise grid-based summaries of 19 “bioclimatic” variables for aspects of temperature and specific humidity (Vega et al., 2017). We eliminated *a priori* mean temperature of most humid quarter, mean temperature of least humid quarter, mean specific humidity of warmest quarter, and mean specific humidity of coldest quarter, because they can contain odd spatial artifacts (Ribeiro et al., 2017, Ashraf et al., 2017).

We then summarized species’ use of these environmental dimensions by means of the values associated with known occurrences of each species; crucially, we also summarized availability of conditions to each species via the values represented across the area hypothesized to be accessible to each species (see above). We assumed that these environmental ranges (use and availability) were continuous, such that we filled all values between maximum and minimum values. In all, we analyzed six layers: annual mean temperature, annual mean specific humidity, the first two principal components of temperature, and the first two principal components of specific humidity.

Environmental data ranges were divided into small bins for analysis (Ribeiro et al., 2017). Specifically, we used 66 bins for temperature and 72 bins for humidity variables. In each bin, we tallied species as present or absent or as unknown. That is, when species were present under extreme or peripheral environmental values within their accessible areas (Owens et al., 2013), we tallied all more extreme (i.e., unavailable) conditions as unknown. Temperature and humidity character bin tables were constructed to be used by the nichevol R package (Cobos et al., 2020; https://github.com/marlonecobos/nichevol) for optimization on ultrametric trees for analysis of niche evolution (R Core Team, 2021). We used reconstruction by both parsimony and likelihood optimization methods (Owens et al., 2013, Owens et al., 2020). The new methodology developed by the Owens et al. (2020) helped to minimize uncertainty in analyzing niche retraction and expansion. We calculated histograms of the species’ environmental ranges for annual mean temperature and mean annual specific humidity (S1 and S2), and used a 95 % confidence level for further analyses in nichevol, which reduces the uncertainty in the analysis.

Ancestral states were reconstructed for each environmental bin on each replicate tree via parsimony methods implemented in Mesquite (version 2.1) (Maddison & Maddison, 2007). Results for individual environmental bins were combined to produce full niche estimates, as the concatenation of the reconstructed results from across all bins for each environmental dimension. Niches were then summarized qualitatively as warm, cold, wet, dry, narrow, and/or broad niches. Final trees were prepared by marking evolutionary changes of these broad categories of niches, and we explored geographic implications of these results by visualizations of their geographic distribution across the range of the family. The basal node of the tree in Fig. 3 of Besnard et al. (2009) was dated at 59 MYA, which was based on 95 % of the posterior distribution of heights for each node; we used the ultrametric tree kindly provided to us by Besnard to date (approximately) subsequent nodes in the diversification of the group.

**Fig. 3:**
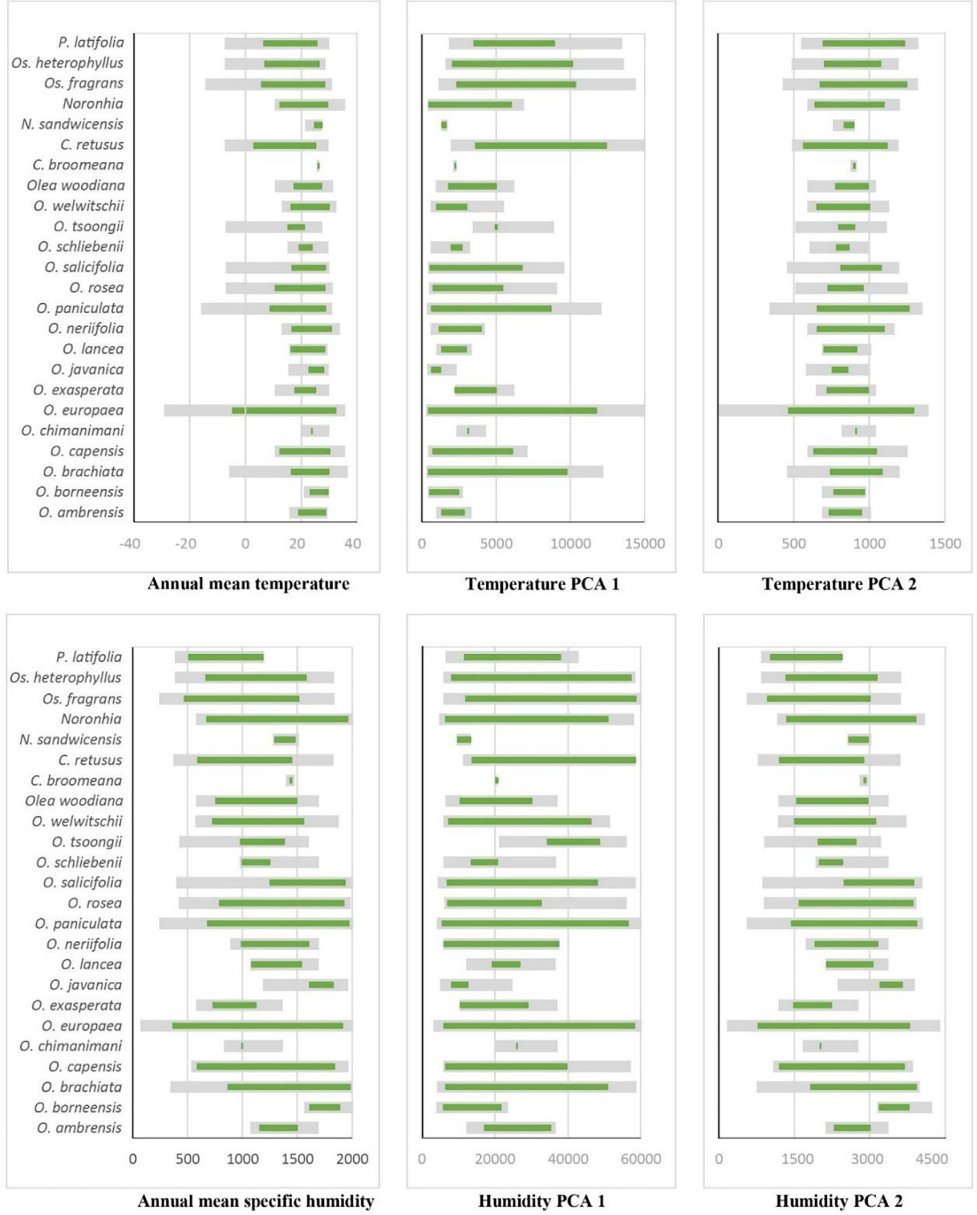
Annual mean temperature and specific humidity for each of 24 species of Oleaceae: environmental ranges for species’ accessible areas (**M**) are shown in gray; realized ecological niches (Soberón, 2007) are shown in green.

## 3 Results

### 3.1 Geographic distributions of species

Current distribution of 24 Oleaceae species and the associated hypothesized accessible geographic areas are shown in Fig. 1, Fig. 2, respectively. The species *Olea europaea* is distributed worldwide in view of its use to produce fruit and as an ornamental plant. Other Oleaceae species are distributed across parts of South Africa, the Mediterranean region, and Asia. Restricted-range species include *O. ambrensis* (Madagascar)*, O. borneensis* (Malaysia and Philippines)*, O. brachiata* (southwestern Asia)*, O. javanica* (Malaysia, Indonesia, Papua, New Guinea, and Cambodia)*, O. lancea* (Madagascar and Mauritius)*,* and *O. rosea* (Yemen, Thailand, and Malaysia)*.*

Visualizations of environmental distributions across the accessible areas for each species are shown in Fig. 3, indicating their realized ecological niches (Soberón, 2007). *Olea europaea* shows the widest environmental range of any of the species, reflecting its broad geographic range, with annual mean temperatures ranging from −4.6 °C to 32.3 °C. Among other species, the lowest minimum temperature values were used by *Chinonanthus retusus* (3.0 °C) and *O. paniculata* (8.8 °C). In terms of maximum temperatures, *O. neriifolia* approached *O. europaea* with 30.9 °C. Some species can tolerate broad temperature ranges (*O. europaea, O. paniculata, Chionanthus retusus, Osmanthus heterophyllus*), whereas others used only narrow temperature ranges (e.g., *O. chimanimani*, *Chionanthus broomeana*).

Annual mean specific humidity represents a second important dimension; *O. europaea* ranged 366–1912 M * kg of water/kg. *Olea brachiata* and *Noronhia* used high-humidity environments, with maximum annual specific humidity of 1983 and 1950 M*kg of water/kg, respectively. Species in less humid environments included *Osmanthus fragrans, Phillyrea latifolia,* and *Olea capensis* (Fig. 3).

### 3.2 Phylogenetic analysis

**Fig. 4:**
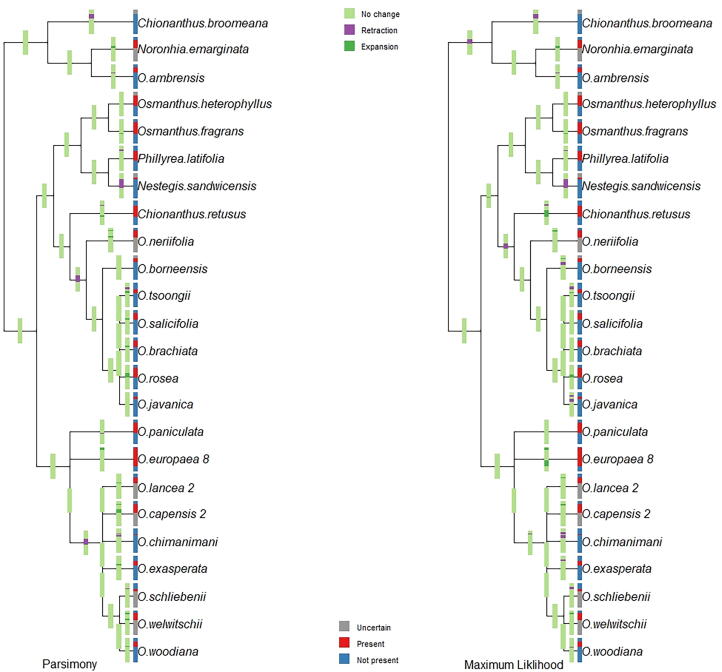
Species’ environmental usage and niche evolution reconstructed through parsimony and maximum likelihood method in term of temperature for 24 species of Oleaceae, based on four plastid DNA regions (*trnT-trnL, trnL-trnF, trnS-try*, and *matK*), termed tree 1.

**Fig. 5:**
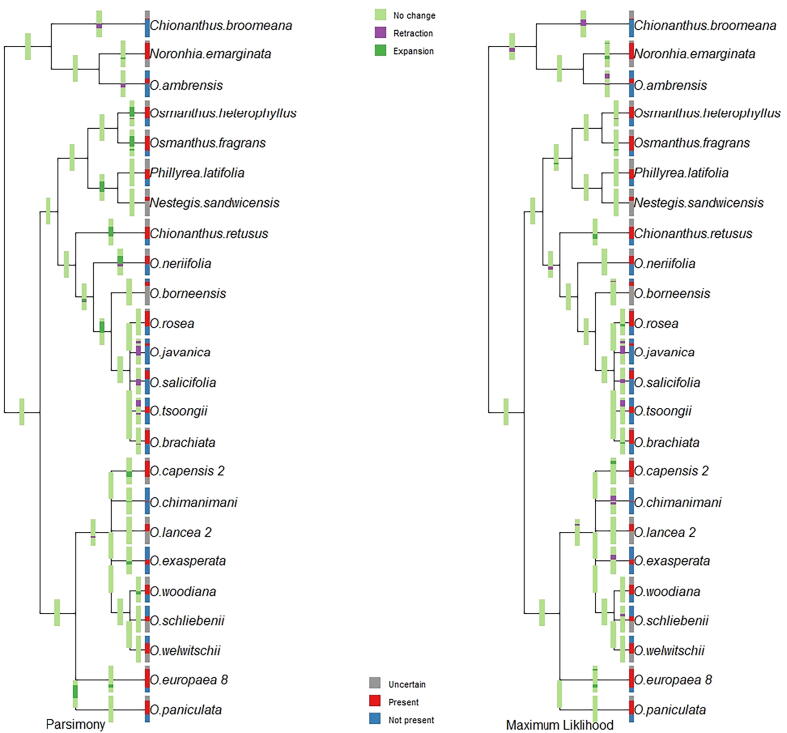
Species’ environmental usage and niche evolution reconstructed through parsimony and maximum likelihood method in term of humidity for 24 species of Oleaceae, based on four plastid DNA regions (*trnT-trnL, trnL-trnF, trnS-try*, and *matK*), termed tree 1.

The original trees used in our phylogenetic analyses (Besnard et al., 2009) included 64 and 53 taxa, based on four plastid DNA (trnT-trnL, trnL-trnF, trnS-try and matK) and nuclear ribosomal DNA, respectively (Fig. 1, Fig. 2, in Besnard et al., 2009). Our initial phylogenetic analyses of niche traits are represented in Fig. 4, Fig. 5 for tree 1 (Supplementary Fig. 1s and 2s for tree 2). Fig. 4, Fig. 5 shows ranges of environmental parameters (temperature and humidity) at each node of the tree, illustrating ancestral states reconstructed with niche expansion and retraction for each node on the tree using both parsimony and likelihood methods (for tree 2, see Fig. 4s and 5s). Topological differences between the trees used are shown in Fig. 5s.

**Fig. 6:**
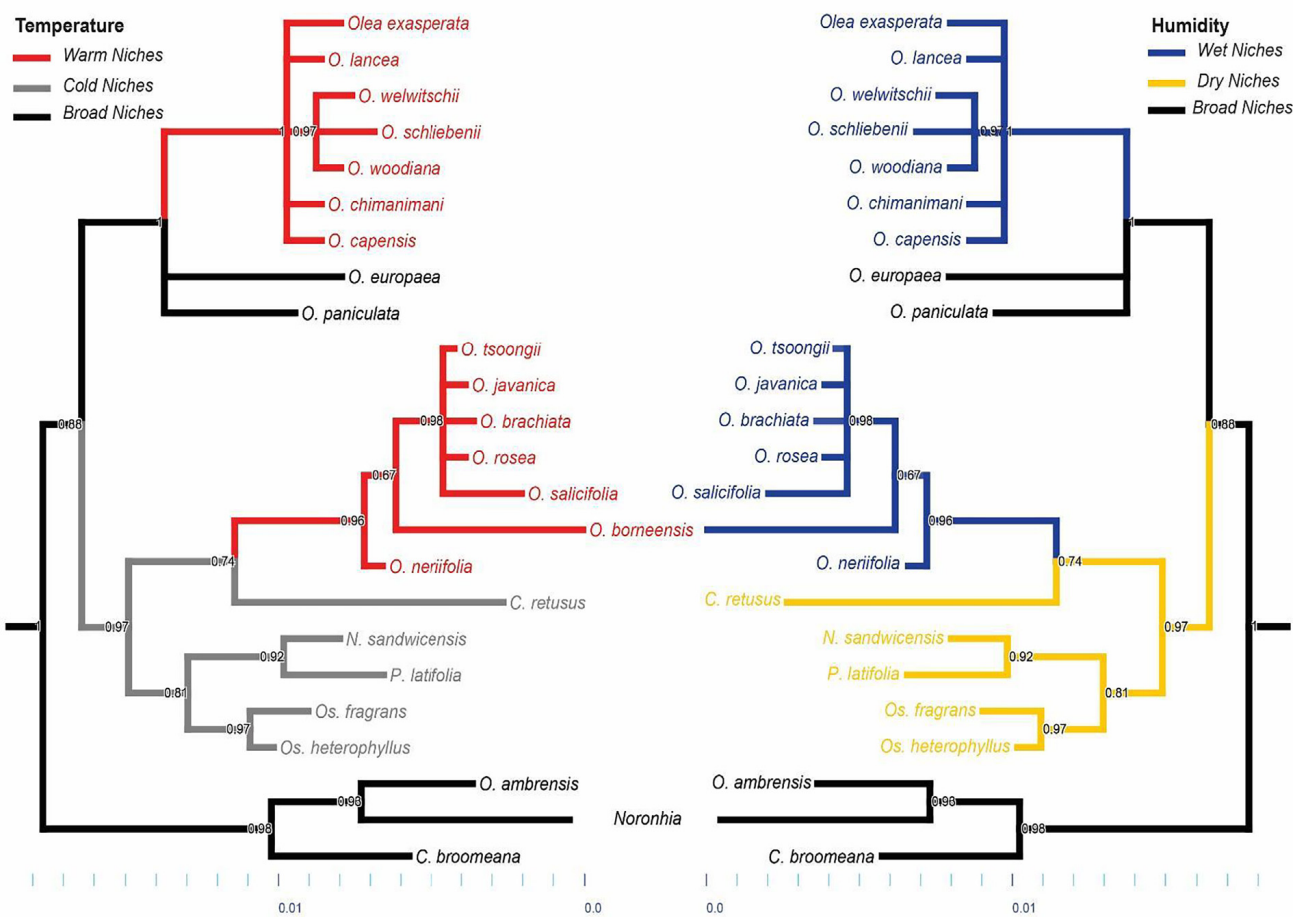
Ancestral state reconstructions in term of broad classes of ecological niches in term of temperature and humidity.

Final ancestral-state reconstructions for major niche characteristics are shown in Fig. 6 for mean annual temperature and mean annual specific humidity for tree 1 (Fig. 3s shows results for tree 2). Environmental ranges were explored for each species via visualizations (histograms) to reduce uncertainty for temperature and humidity (see supplementary material S1 and S2). For temperature, niches were classified as warm or cold; for humidity, niches were divided into wet versus dry niches; niches were also divided into broad versus narrow niches. *Olea lancea, O. welwitschii, O. woodiana, O. chimanimani, O. tsoongii, O. javanica, O. brachiata, O. rosea, O. salicifolia, O. borneensis,* and *O. neriifolia* had warm niches; *Chionanthus retusus, Phillyrea latifolia, Osmanthus fragrans*, and *O. heterophyllus* had cold and dry niches; *Olea tsoongii, O. javanica, O. brachiata, O. rosea,* and *O. salicifolia* had wet niches; *O. exasperata, O. schliebenii,* and *O. neriifolia* had wet niches; and *O. europaea, O. paniculata, O. ambrensis, Noronhia,* and *Chionanthus broomeana* had broad niches. In general, in both trees, species with warm and wet niches had ancestors with cold and dry niches.

**Fig. 7:**
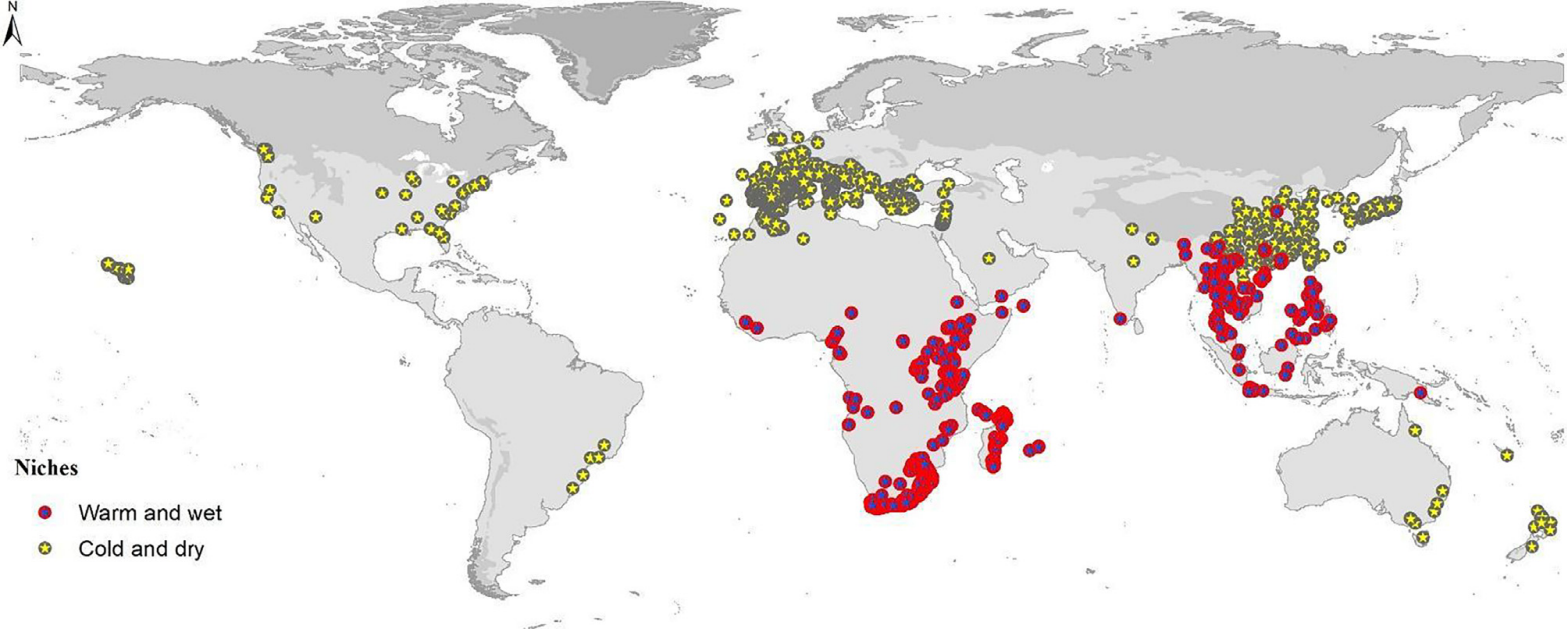
Geographic representation of reconstructed ecological niches for 19 species of Oleaceae, with niches separated into two general groups.

The geographic representation of these niches is shown in Fig. 7 (Fig. 4s for tree 2). *Olea europaea,* with its broad niche is distributed worldwide. Other than that species, Oleaceae species in Africa and Southeast Asia had both warm and wet niches, and cold and dry niches respectively; southeastern Australia and the Mediterranean region hold species that share cold and dry niches. According to our dating based on the analyses of Besnard et al. (2009), main divergence in the species of genus *Olea* from *Nestegis*, *Osmanthus* and *Phillyrea* occurred at ∼ 46 MYA. Niche expansion occurred ∼ 43 MYA for Oleaceae species present on the mainland (Asia, Europe and USA) and niche retraction occurred ∼ 32–35 MYA particularly species present on islands (Madagascar, Reunion, Mauritia’s, Malaysia, Philippines etc.) and southern Africa. Recent niche expansion and retraction (<26 MYA) events irrespective of time happened in different areas depending upon the recent climatic events.

## 4 Discussion

Molecular dating analysis have shown that major climatic events on historical time scales are associated with the diversification in evolving lineages (Douady et al., 2003, Renner, 2016). Niche characteristics, however, usually remain consistent over long time scales in many lineages, and exhibit a slow diversification process over phylogeny (Silva et al., 2020). In this study, we sought to estimate and understand the dynamics of niche characteristics in wild olives and relatives. Our preliminary analysis showed no significant effect on results by removing one of the duplicate values in the tree by randomization. In both, replicated choices were made in *O. europea* (22 sub-species), *O. lancea* (3 sub-species) and *O. capensis* (8 sub-species), which helped to harmonize the two trees. Despite some topological changes in the trees (Fig. 5s), we got similar results for both trees, which indicate the robustness of the used methodology.

Previous phylogenetic studies of the Oleaceae were carried out less comprehensively, or were focused on subclades only (Besnard and Bervillé, 2000, Besnard et al., 2001, Besnard et al., 2007, Besnard et al., 2009, Besnard et al., 2011, Besnard et al., 2013), so this study was the first to include a relatively broad sample of the diversity of the family. Our results give an idea of how clades evolved over time, which lineages share similar niches, and which niche characteristics evolved when in the history of the family, but are subject to all of the caveats and cautions involved in interpretation of incomplete taxon representation in ancestral state reconstructions (Heath et al., 2008). The ancestral state reconstructions for environmental variables helps to identify species that need conservation in the future, as well as to identify the behavior of species in the environmental spaces.

An important feature of our analyses is that we have taken special care to identify sectors of environmental space in which information gaps dominate. That is, we have documented elsewhere that existing niches are a subset of fundamental niches (Peterson et al., 2011), and that reconstructing evolutionary patterns based on existing niches will almost always overestimate amount of evolutionary change (Peterson, 2011, Saupe et al., 2017, Owens et al., 2020). Given that full estimates of fundamental niches are difficult (Peterson et al., 2015), here, we explore the alternative approach of explicit incorporation of knowledge gaps in information available, which allows avoidance of overestimation of amounts of evolutionary change. Our analyses of temperature and humidity use by species gives information about their current abiotic niches (Fig. 3, Soberón, 2007). However, these niche characteristics depend on the completeness of the occurrence data; as such, if sample sizes are small, the result may not be a full representation of the species’ environmental potential. Although we georeferenced many occurrences to increase sample sizes, several species still had small sample sizes, and therefore had small ranges of temperature and humidity (e.g., *Olea chimanimani* and *Chionanthus broomeana*); in many cases, small sample sizes are an unavoidable consequence of microendemic geographic distributions. *Olea europaea,* on the other hand, had the broadest (indeed invasive) geographic distribution and associated ecological niche; its importance in fruit production and as an ornamental is a major factor in its global distribution (Besnard et al., 2007).

Our phylogenetic analysis showed that niche evolution takes place at different nodes of the phylogeny with respect to annual mean temperature and specific humidity. Initially, cold and dry niches evolved from ancestral niches in results of the parsimony and likelihood ancestral-state reconstruction methods. Thereafter, evolutionary change in this group has been in a process of evolving toward warm and wet niches, in particular among lineages in Southeast Asia. Warm and narrow niches evolved before warm and wet niches. In the time-referenced tree, cold and dry niches appeared to have evolved ∼ 46 MYA, and warm and wet niches evolved in the interval ∼ 40 to 34 MYA. High temperature in Lutetian (Oligocene) and low temperature in Rupelian (Eocene) with major desertification events play important role for niche retraction and expansion in the history for Oleaceae clades. However, no niche evolution apparently occurred in *Olea ambrensis*, *Chionanthus broomeana*, and *Noronhia* thanks to their broader niches. Niche evolution analysis illustrates that uncertainty may increases in the parsimony reconstruction analysis in comparison with maximum likelihood methods (Owens et al., 2020).

Focusing on maximum likelihood analysis, niche expansion at both ends (colder and warmer) in *O. europaea* is clearly associated with its recent economic importance, as many olive cultivars likely contribute to its broad temperature and humidity tolerance (T= ∼ −4.6 °C to 32 °C and H = 366 to 1912 M * kg of water/kg). Warming processes near the end of Pleistocene brought a reticulation event in the *O. europaea* species complex (Besnard et al., 2007 & 2009). Niche retractions can be seen in *Chionanthus broomeana, O. borneensis, O. tsoongi, O. Javanica, O. chimanimani* and *O. exasperata* species (Fig. 4, Fig. 5). Reticulation events play an important role in evolution between the lineages of this clade with different niche expansion and retraction events in different clades and nodes (Robio de Casas et al., 2006, Besnard et al., 2009). The association between niche characteristics and phylogeny reconstruction plays an important role to understand fundamental ecological questions related to speciation.

Overall, we found considerable phylogenetic niche conservatism in the Oleaceae despite having broad geographic spread in species in the family. Although we could not make strong comments regarding other genera of Oleaceae owing to low numbers of species in the tree. Here we also identify niche retraction indicating species that need to be conserved in future climates in view of their retracting niches and consequently reduced distributional potential.

## 5 Significance statement

Climate is an important parameter in delimiting coarse-grained aspects of fundamental ecological niches of species and their evolution is key in biological diversification. The olive family is both diverse and broadly distributed geographically, yet ecological niche evolution has not been assessed in this group. Our analysis revealed relatively slow or conservative niche evolution in this group and explores how the geographic potential of the group has responded to evolutionary changes in those niches.

## Declaration of Competing Interest

The authors declare that they have no known competing financial interests or personal relationships that could have appeared to influence the work reported in this paper.
