# Supplementary material for: Global ecological niche conservatism and evolution in Olea species
Source: Saudi J Biol Sci. 2022 Nov 11;30(1):103500. doi: 10.1016/j.sjbs.2022.103500 (PMC9706617; doi:10.1016/j.sjbs.2022.103500)
Supplement: Supplementary figures and data [file mmc1.zip › S1-Histograms_bio1.pdf]

Values and median deviations for variable bio1

Description

The information presented below visualizes the distribution of variable values in the accessible area, as well as the species occurrences, to facilitate the delimitation of conditions to be used in further analyses.

Symbology

- Occurrences
- 95% Confidence limits
- 99% Confidence limits

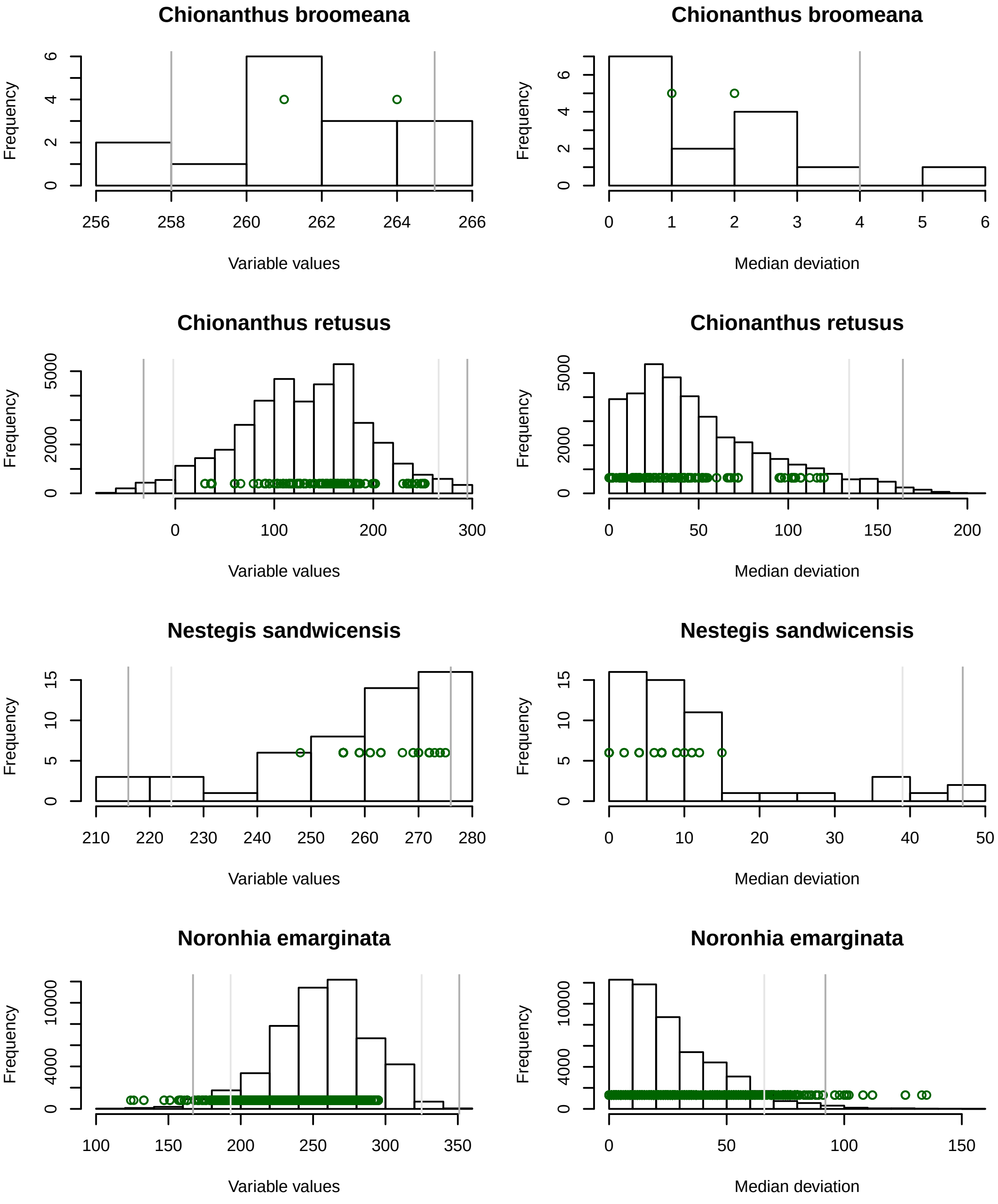

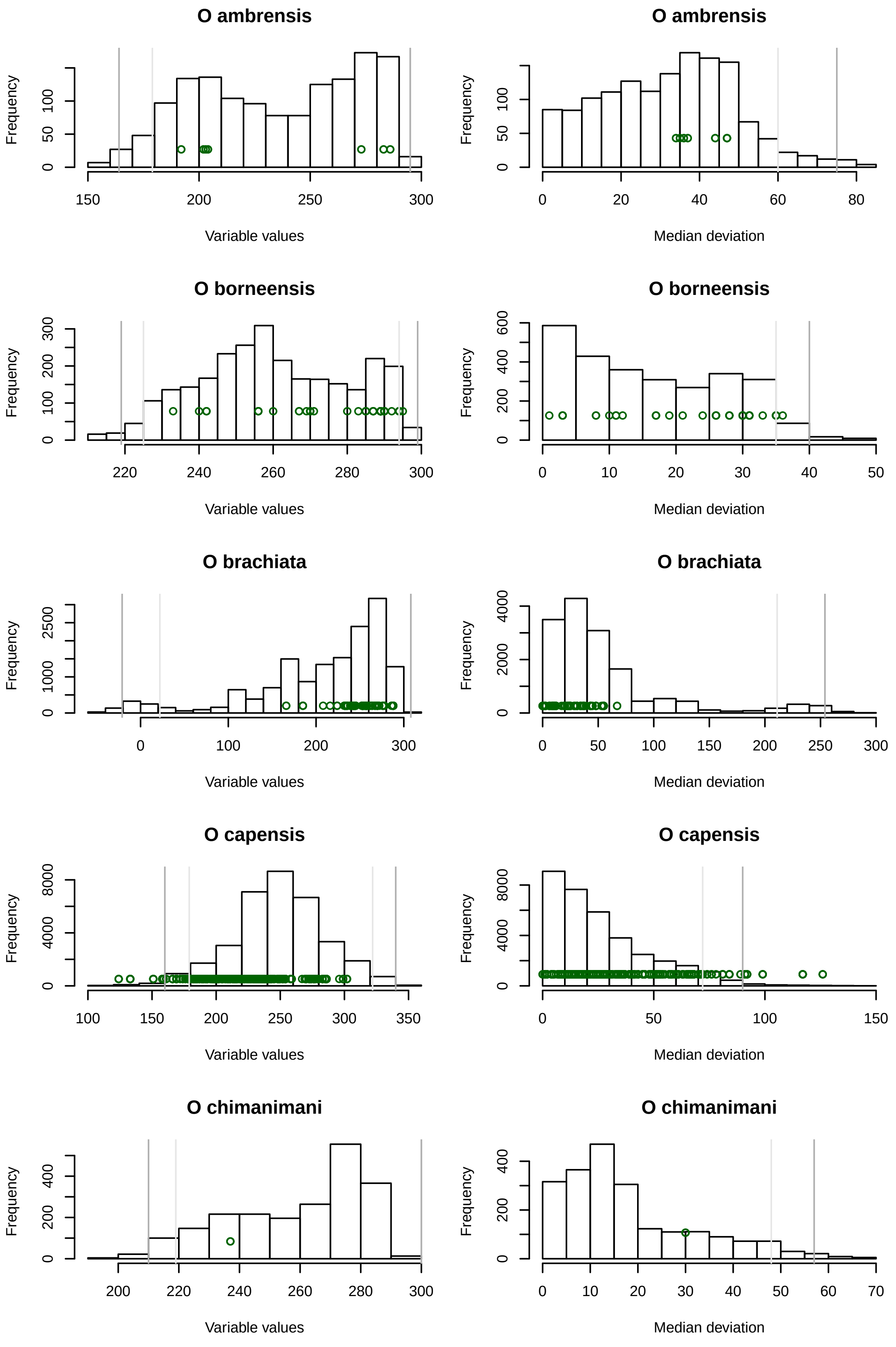

O ambrensis
Frequency
Variable values
O ambrensis
Frequency
Median deviation
O borneensis
Frequency
Variable values
O borneensis
Frequency
Median deviation
O brachiata
Frequency
Variable values
O brachiata
Frequency
Median deviation
O capensis
Frequency
Variable values
O capensis
Frequency
Median deviation
O chimanimani
Frequency
Variable values
O chimanimani
Frequency
Median deviation

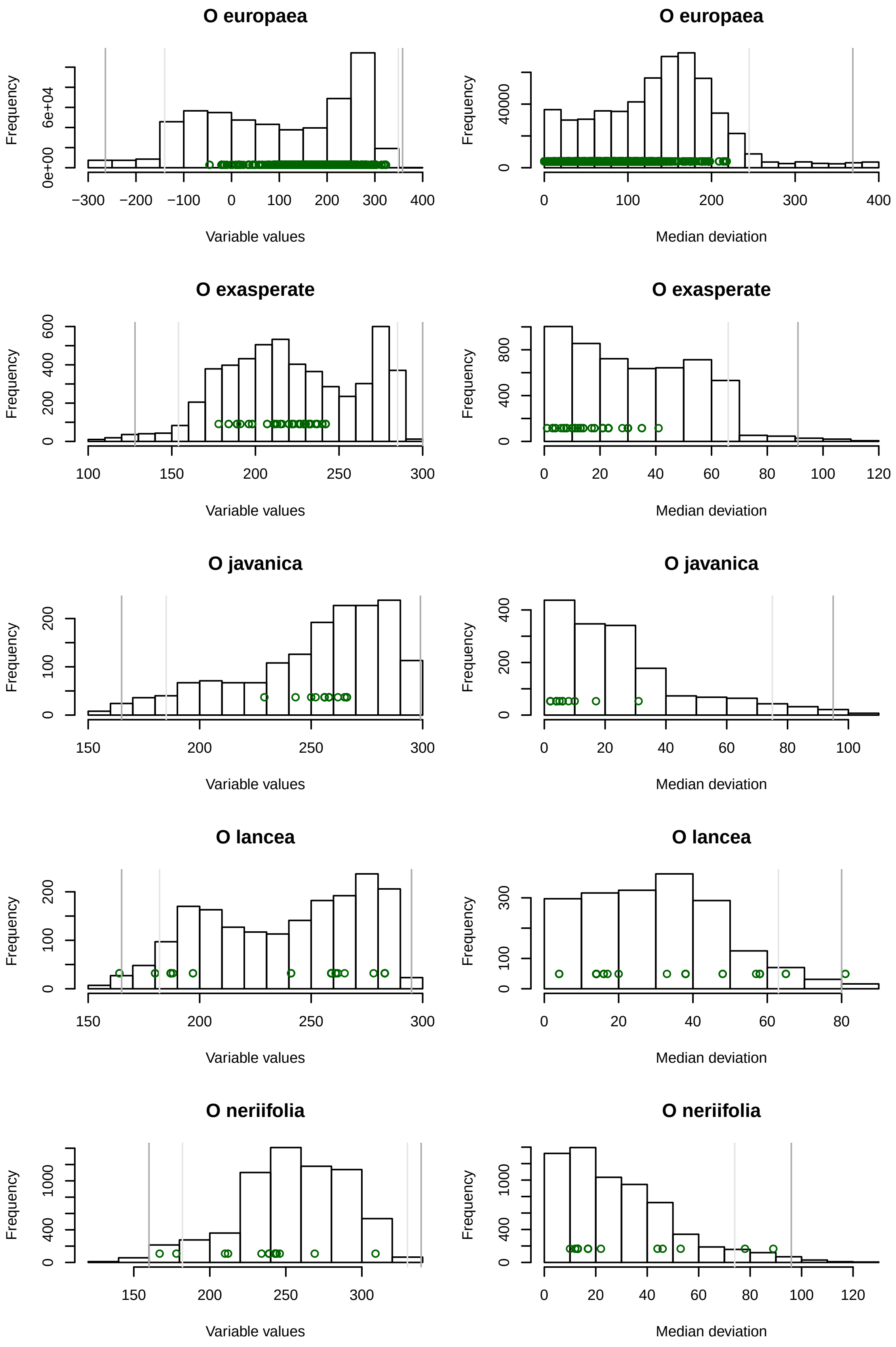

O europaea
Frequency
0e+00
6e+04
−300
−200
−100
0
100
200
300
400
Variable values
O europaea
Frequency
0
40000
0
100
200
300
400
Median deviation
O exasperate
Frequency
0
200
400
600
100
150
200
250
300
Variable values
O exasperate
Frequency
0
400
800
0
20
40
60
80
100
120
Median deviation
O javanica
Frequency
0
100
200
150
200
250
300
Variable values
O javanica
Frequency
0
200
400
0
20
40
60
80
100
Median deviation
O lancea
Frequency
0
100
200
150
200
250
300
Variable values
O lancea
Frequency
0
100
300
0
20
40
60
80
Median deviation
O neriifolia
Frequency
0
400
1000
150
200
250
300
Variable values
O neriifolia
Frequency
0
400
1000
0
20
40
60
80
100
120
Median deviation

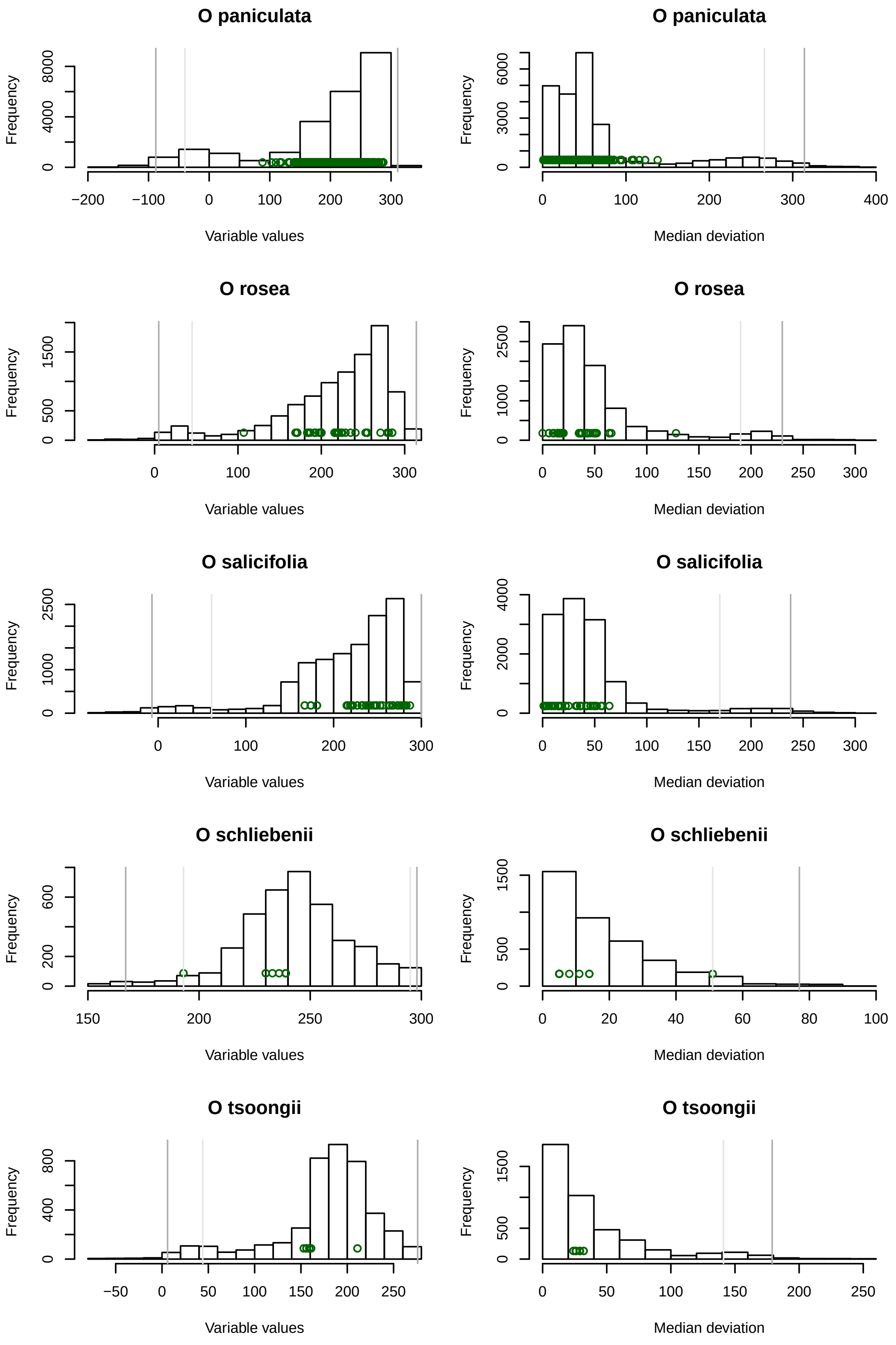
O paniculata
Frequency
0
4000
8000
−200
−100
0
100
200
300
Variable values
O paniculata
Frequency
0
3000
6000
0
100
200
300
400
Median deviation
O rosea
Frequency
0
500
1500
0
100
200
300
Variable values
O rosea
Frequency
0
1000
2500
0
50
100
150
200
250
300
Median deviation
O salicifolia
Frequency
0
1000
2500
0
100
200
300
Variable values
O salicifolia
Frequency
0
2000
4000
0
50
100
150
200
250
300
Median deviation
O schliebenii
Frequency
0
200
600
150
200
250
300
Variable values
O schliebenii
Frequency
0
500
1500
0
20
40
60
80
100
Median deviation
O tsoongii
Frequency
0
400
800
−50
0
50
100
150
200
250
Variable values
O tsoongii
Frequency
0
500
1500
0
50
100
150
200
250
Median deviation

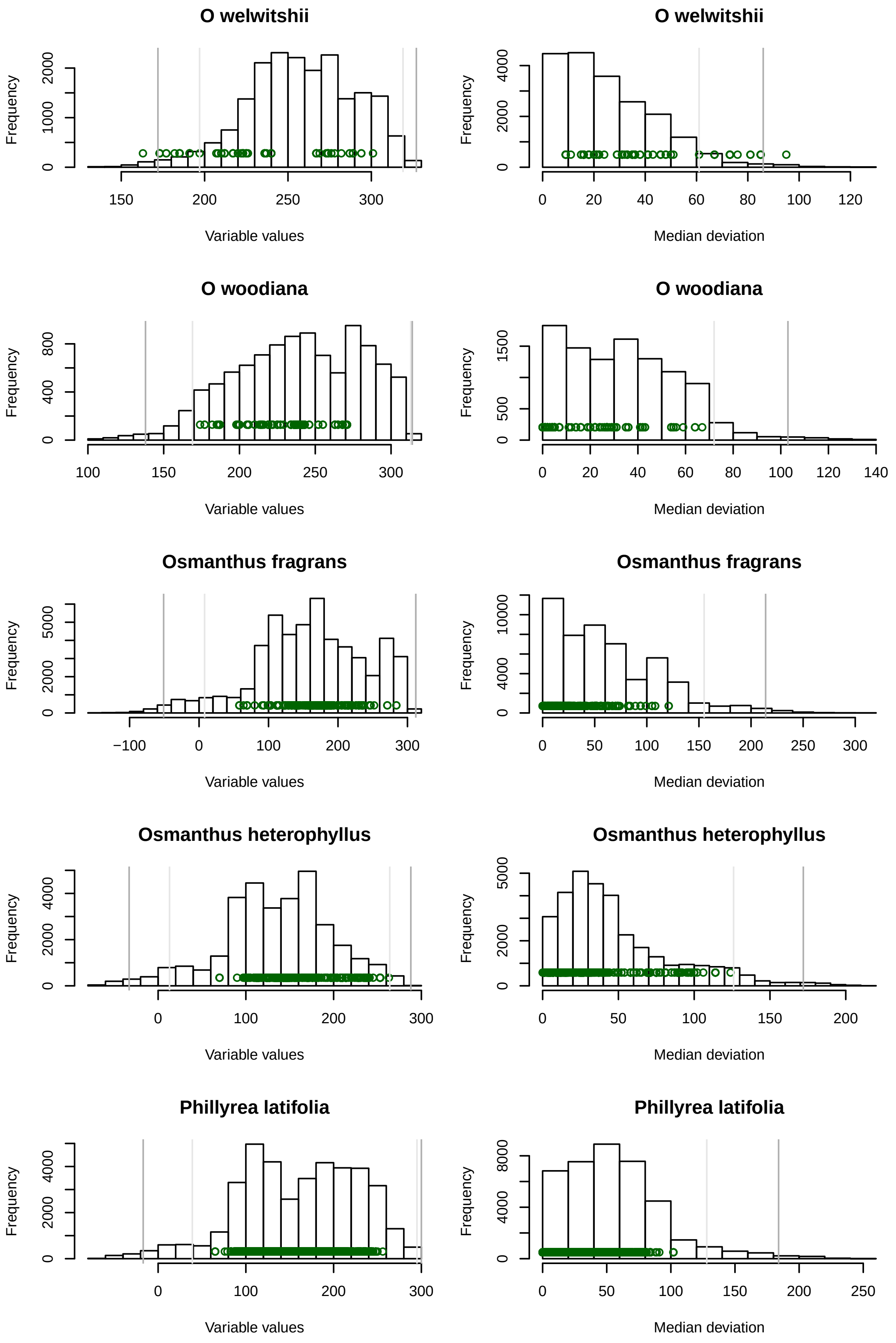
O welwitshii
Frequency
0
1000
2000
150
200
250
300
Variable values
O welwitshii
Frequency
0
2000
4000
0
20
40
60
80
100
120
Median deviation
O woodiana
Frequency
0
400
800
100
150
200
250
300
Variable values
O woodiana
Frequency
0
500
1500
0
20
40
60
80
100
120
140
Median deviation
Osmanthus fragrans
Frequency
0
2000
5000
−100
0
100
200
300
Variable values
Osmanthus fragrans
Frequency
0
4000
10000
0
50
100
150
200
250
300
Median deviation
Osmanthus heterophyllus
Frequency
0
2000
4000
0
100
200
300
Variable values
Osmanthus heterophyllus
Frequency
0
2000
5000
0
50
100
150
200
Median deviation
Phillyrea latifolia
Frequency
0
2000
4000
0
100
200
300
Variable values
Phillyrea latifolia
Frequency
0
4000
8000
0
50
100
150
200
250
Median deviation
