# Supplementary material for: Global ecological niche conservatism and evolution in Olea species
Source: Saudi J Biol Sci. 2022 Nov 11;30(1):103500. doi: 10.1016/j.sjbs.2022.103500 (PMC9706617; doi:10.1016/j.sjbs.2022.103500)
Supplement: Supplementary figures and data [file mmc1.zip › S2-Histograms_bio12.pdf]

Values and median deviations for variable bio12

Description

The information presented below visualizes the distribution of variable values in the accessible area, as well as the species occurrences, to facilitate the delimitation of conditions to be used in further analyses.

Symbology

- Occurrences
- 95% Confidence limits
- 99% Confidence limits

Chionanthus broomeana

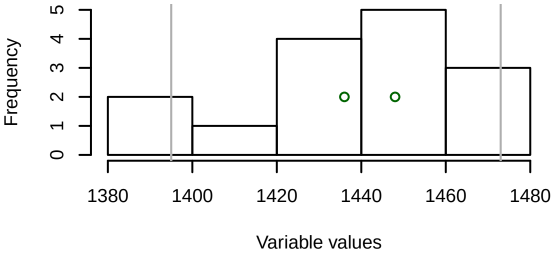

Chionanthus broomeana

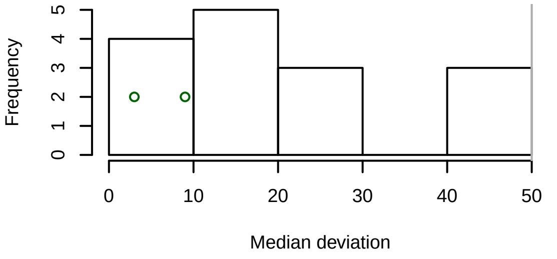

Chionanthus retusus

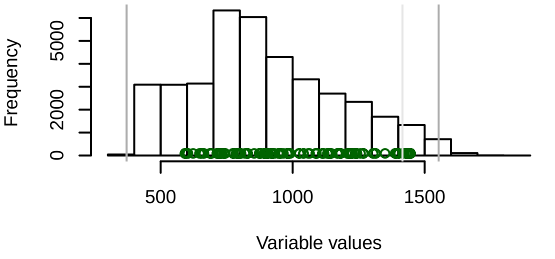

Chionanthus retusus

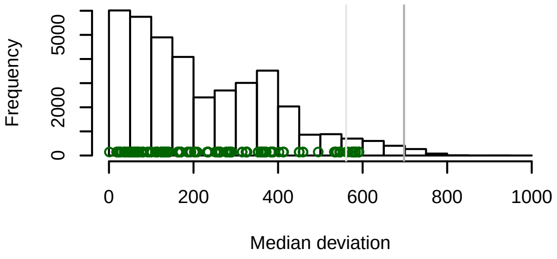

Nestegis sandwicensis

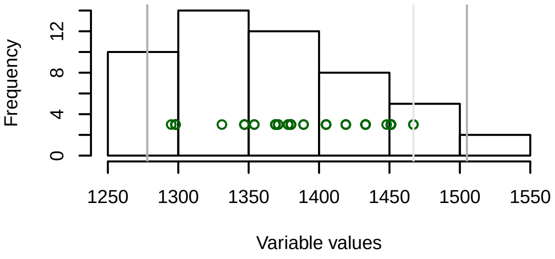

Nestegis sandwicensis

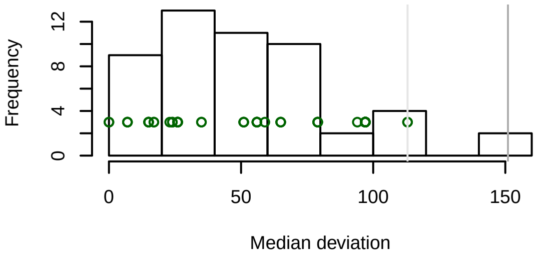

Noronhia emarginata

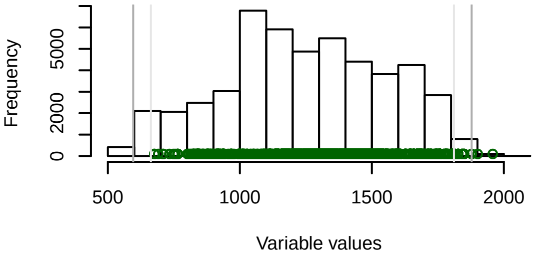

Noronhia emarginata

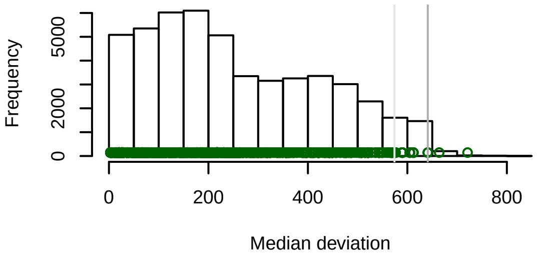

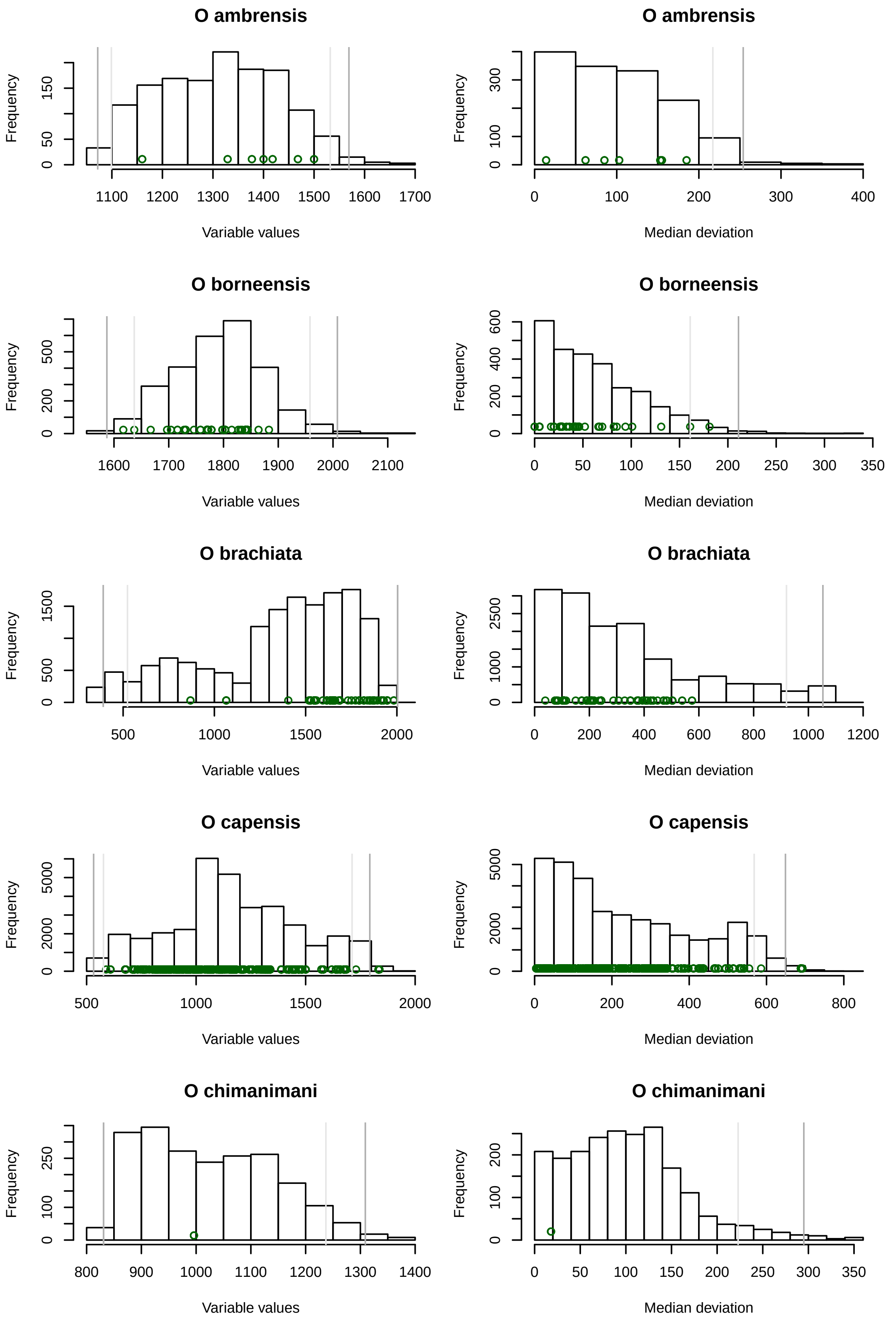
O ambrensis
Frequency
0
50
150
1100
1200
1300
1400
1500
1600
1700
Variable values
O ambrensis
Frequency
0
100
300
0
100
200
300
400
Median deviation
O borneensis
Frequency
0
200
500
1600
1700
1800
1900
2000
2100
Variable values
O borneensis
Frequency
0
200
400
600
0
50
100
150
200
250
300
350
Median deviation
O brachiata
Frequency
0
500
1500
500
1000
1500
2000
Variable values
O brachiata
Frequency
0
1000
2500
0
200
400
600
800
1000
1200
Median deviation
O capensis
Frequency
0
2000
5000
500
1000
1500
2000
Variable values
O capensis
Frequency
0
2000
5000
0
200
400
600
800
Median deviation
O chimanimani
Frequency
0
100
250
800
900
1000
1100
1200
1300
1400
Variable values
O chimanimani
Frequency
0
100
200
0
50
100
150
200
250
300
350
Median deviation

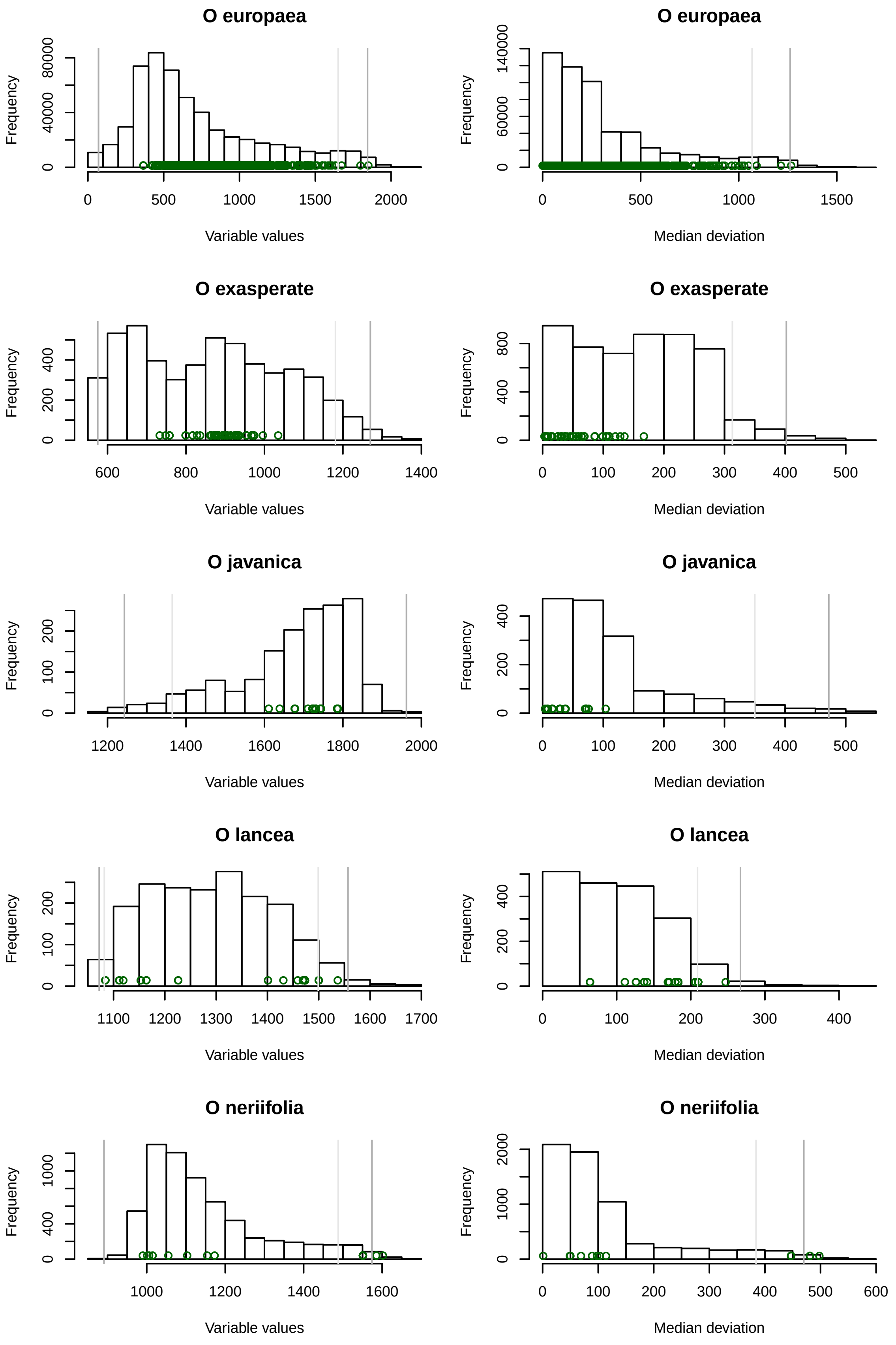

O europaea
Frequency
0
40000
80000
0
500
1000
1500
2000
Variable values
O europaea
Frequency
0
60000
140000
0
500
1000
1500
Median deviation
O exasperate
Frequency
0
200
400
600
800
1000
1200
1400
Variable values
O exasperate
Frequency
0
400
800
0
100
200
300
400
500
Median deviation
O javanica
Frequency
0
100
200
1200
1400
1600
1800
2000
Variable values
O javanica
Frequency
0
200
400
0
100
200
300
400
500
Median deviation
O lancea
Frequency
0
100
200
1100
1200
1300
1400
1500
1600
1700
Variable values
O lancea
Frequency
0
200
400
0
100
200
300
400
Median deviation
O neriifolia
Frequency
0
400
1000
1000
1200
1400
1600
Variable values
O neriifolia
Frequency
0
1000
2000
0
100
200
300
400
500
600
Median deviation

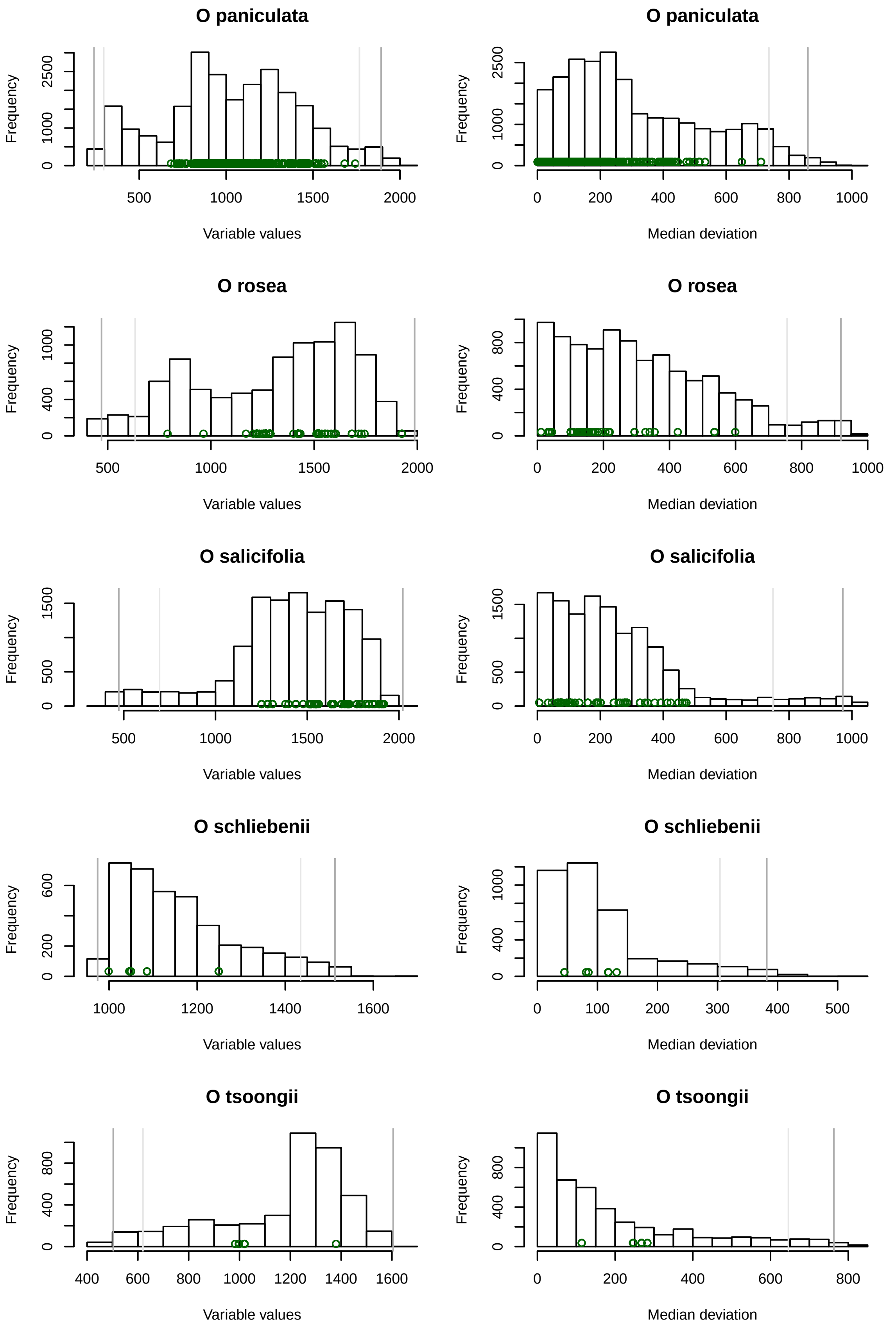
O paniculata
Frequency
0
1000
2500
500
1000
1500
2000
Variable values
O paniculata
Frequency
0
1000
2500
0
200
400
600
800
1000
Median deviation
O rosea
Frequency
0
400
1000
500
1000
1500
2000
Variable values
O rosea
Frequency
0
400
800
0
200
400
600
800
1000
Median deviation
O salicifolia
Frequency
0
500
1500
500
1000
1500
2000
Variable values
O salicifolia
Frequency
0
500
1500
0
200
400
600
800
1000
Median deviation
O schliebenii
Frequency
0
200
600
1000
1200
1400
1600
Variable values
O schliebenii
Frequency
0
400
1000
0
100
200
300
400
500
Median deviation
O tsoongii
Frequency
0
400
800
400
600
800
1000
1200
1400
1600
Variable values
O tsoongii
Frequency
0
400
800
0
200
400
600
800
Median deviation

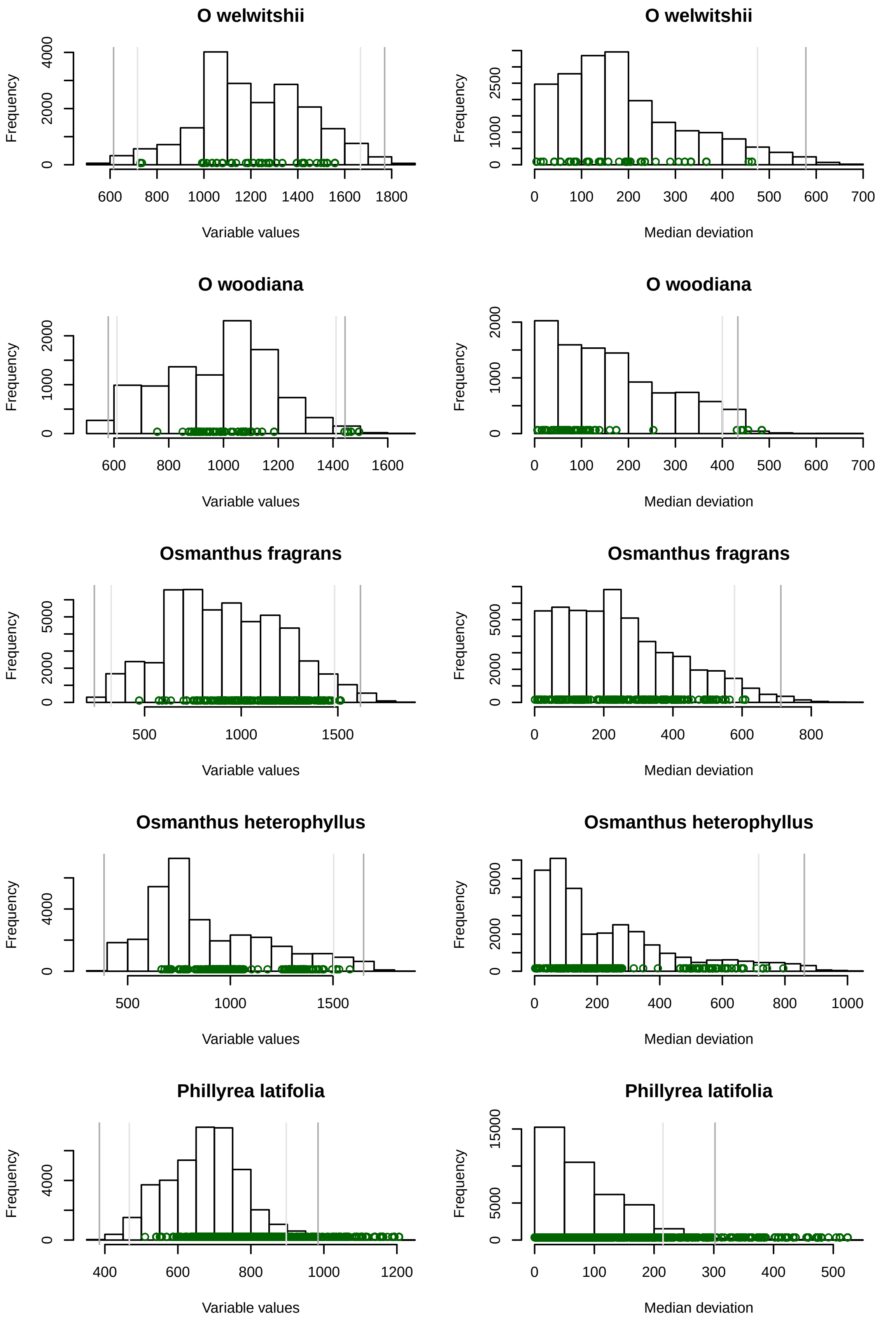
O welwitshii
Frequency
0
2000
4000
600
800
1000
1200
1400
1600
1800
Variable values
O welwitshii
Frequency
0
1000
2500
0
100
200
300
400
500
600
700
Median deviation
O woodiana
Frequency
0
1000
2000
600
800
1000
1200
1400
1600
Variable values
O woodiana
Frequency
0
1000
2000
0
100
200
300
400
500
600
700
Median deviation
Osmanthus fragrans
Frequency
0
2000
5000
500
1000
1500
Variable values
Osmanthus fragrans
Frequency
0
2000
5000
0
200
400
600
800
Median deviation
Osmanthus heterophyllus
Frequency
0
4000
500
1000
1500
Variable values
Osmanthus heterophyllus
Frequency
0
2000
5000
0
200
400
600
800
1000
Median deviation
Phillyrea latifolia
Frequency
0
4000
400
600
800
1000
1200
Variable values
Phillyrea latifolia
Frequency
0
5000
15000
0
100
200
300
400
500
Median deviation
