# Supplementary material for: Global ecological niche conservatism and evolution in Olea species
Source: Saudi J Biol Sci. 2022 Nov 11;30(1):103500. doi: 10.1016/j.sjbs.2022.103500 (PMC9706617; doi:10.1016/j.sjbs.2022.103500)
Supplement: Supplementary figures and data [file mmc1.zip › Supplementary Figures.docx]

**Title: Global ecological niche conservatism and evolution in *Olea* species**

**Supplementary Material**


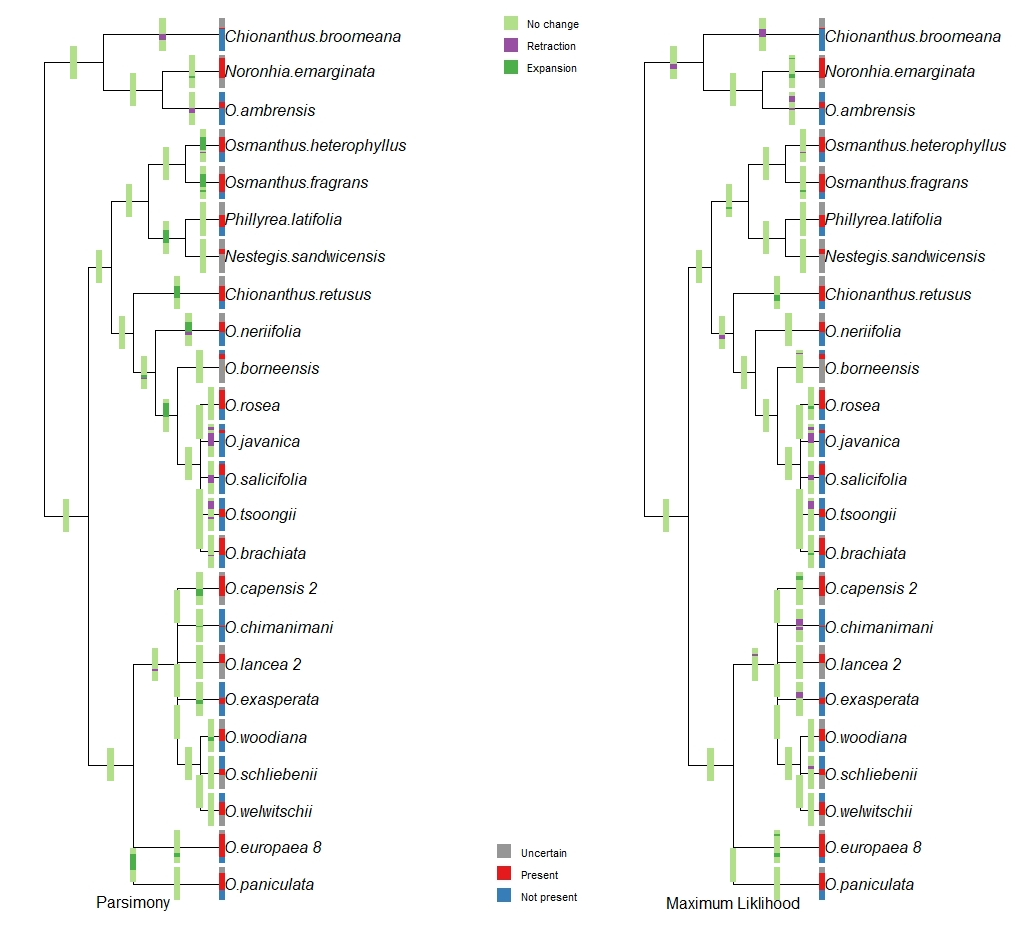


Figure 1s: Species’ environmental usage and niche evolution reconstructed through parsimony and maximum likelihood method in term of temperature for 24 species of Oleaceae, based on nuclear ribosomal DNA (*ITS-1*), termed tree 2


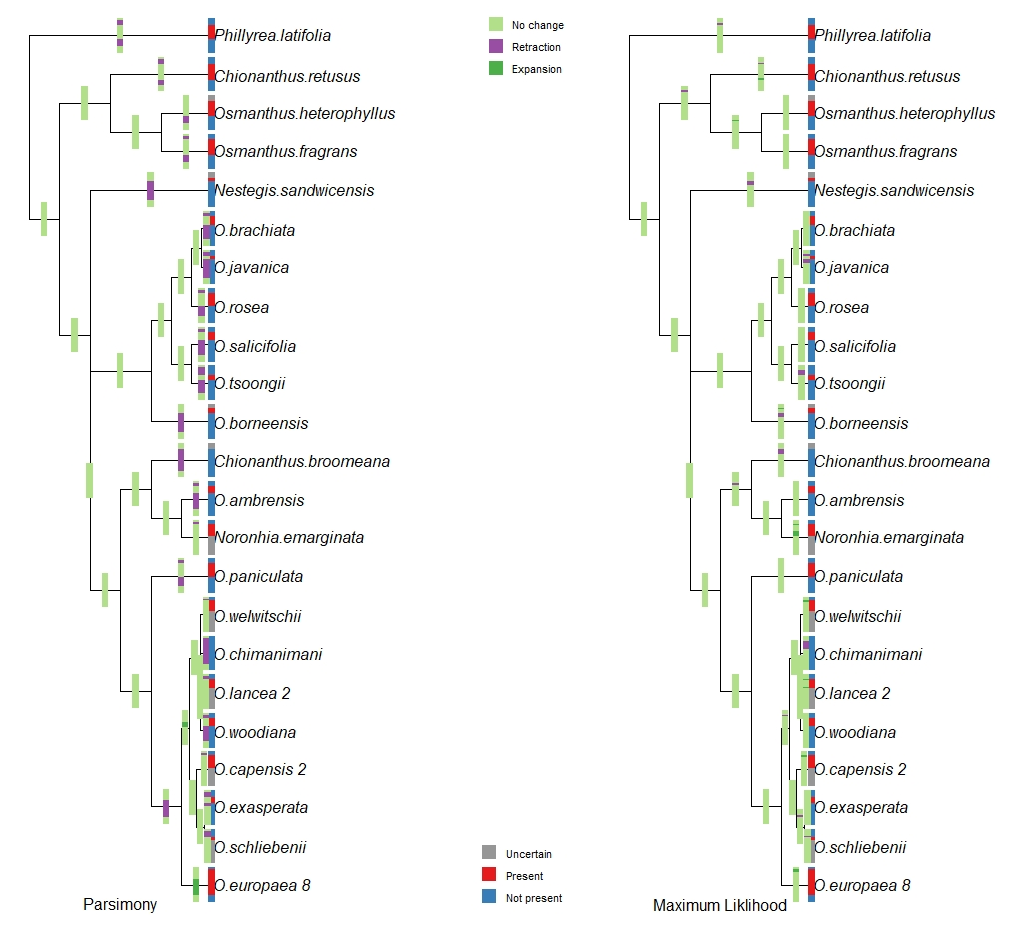


Figure 2s: Species’ environmental usage and niche evolution reconstructed through parsimony and maximum likelihood method in term of humidity for 24 species of Oleaceae, based on nuclear ribosomal DNA (ITS-1), termed tree 2


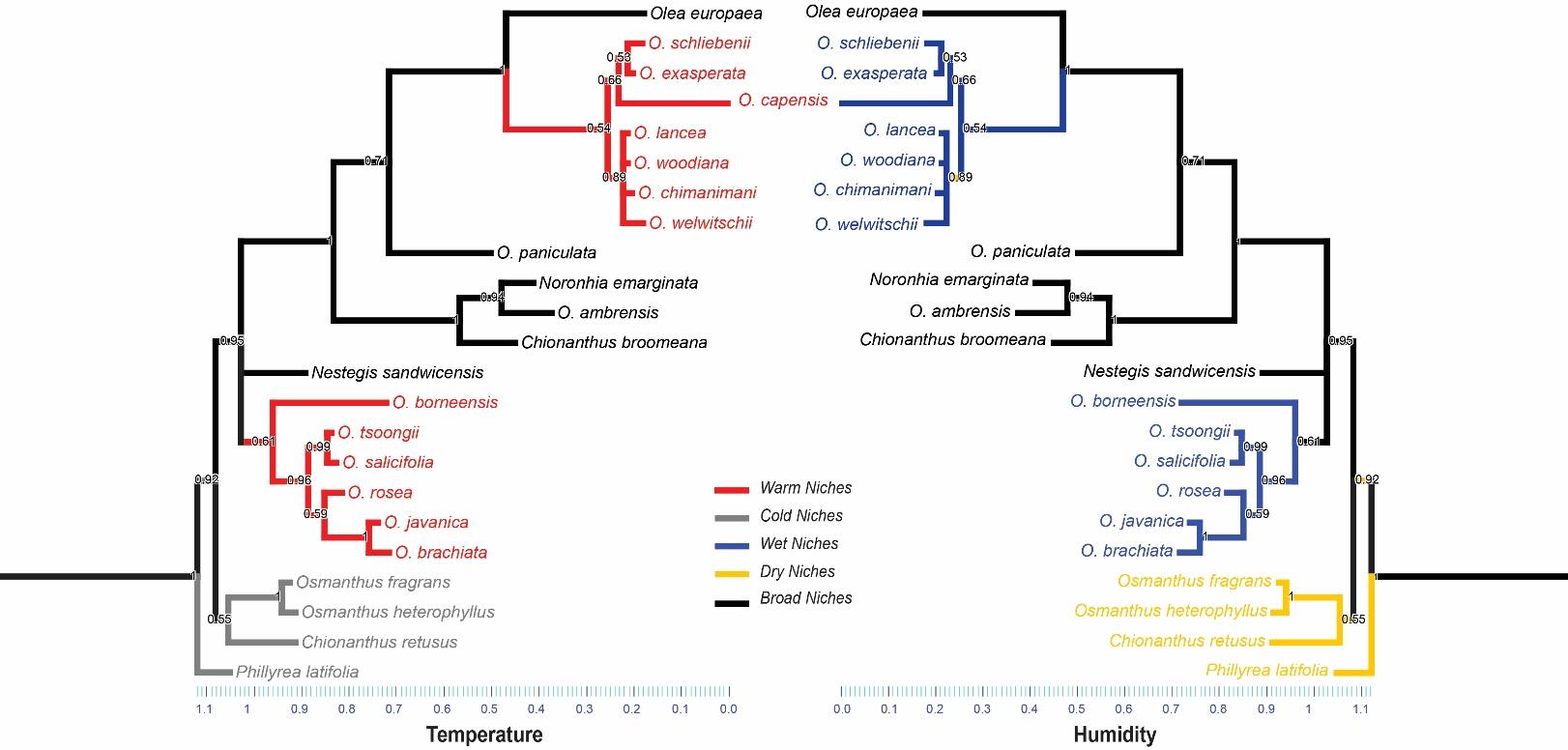


*Figure 3s:* Ancestral state reconstructions in term of broad classes of ecological niches in term of temperature and humidity based on tree 2


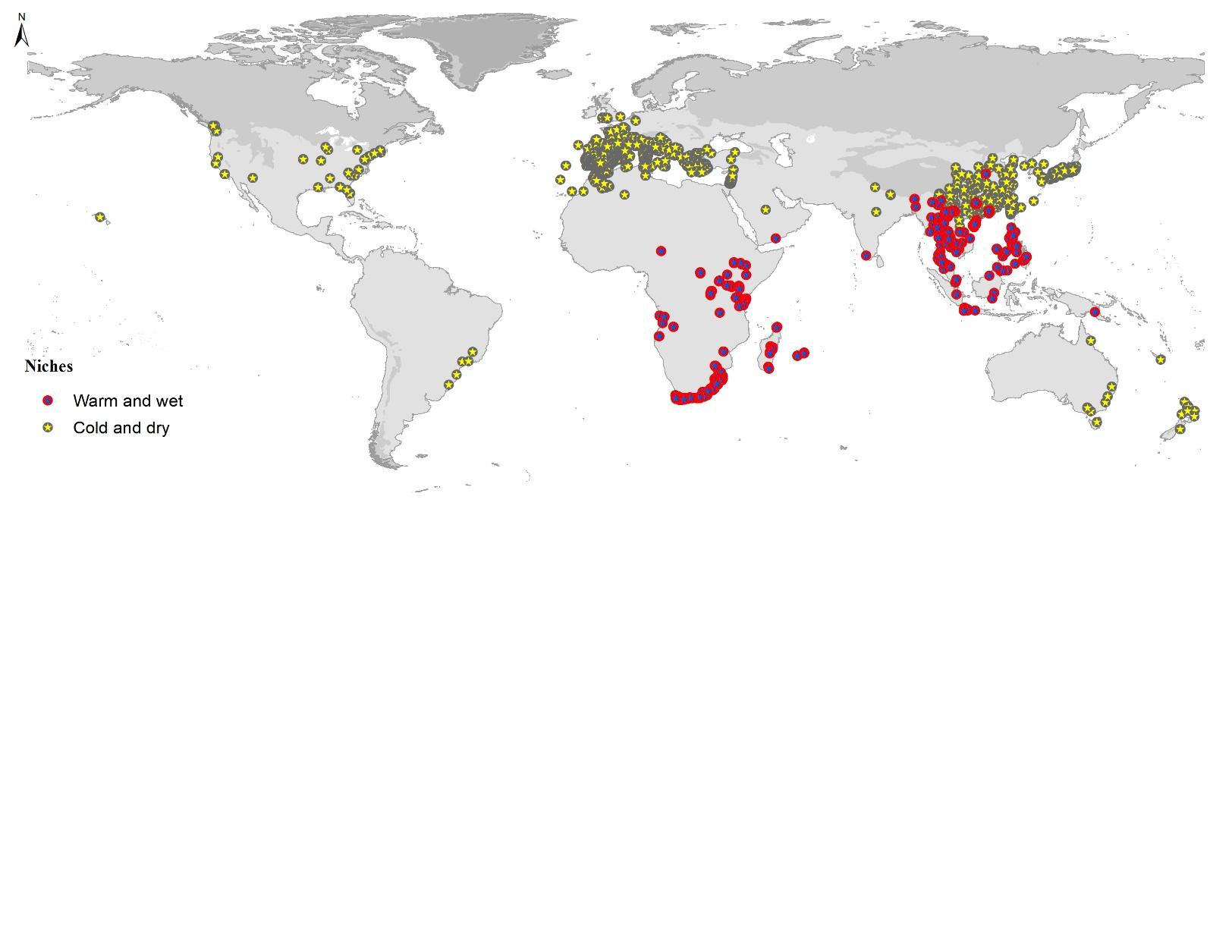


*Figure 4s:* Geographic representation of reconstructed ecological niches for 24 species of Oleaceae based on tree 2


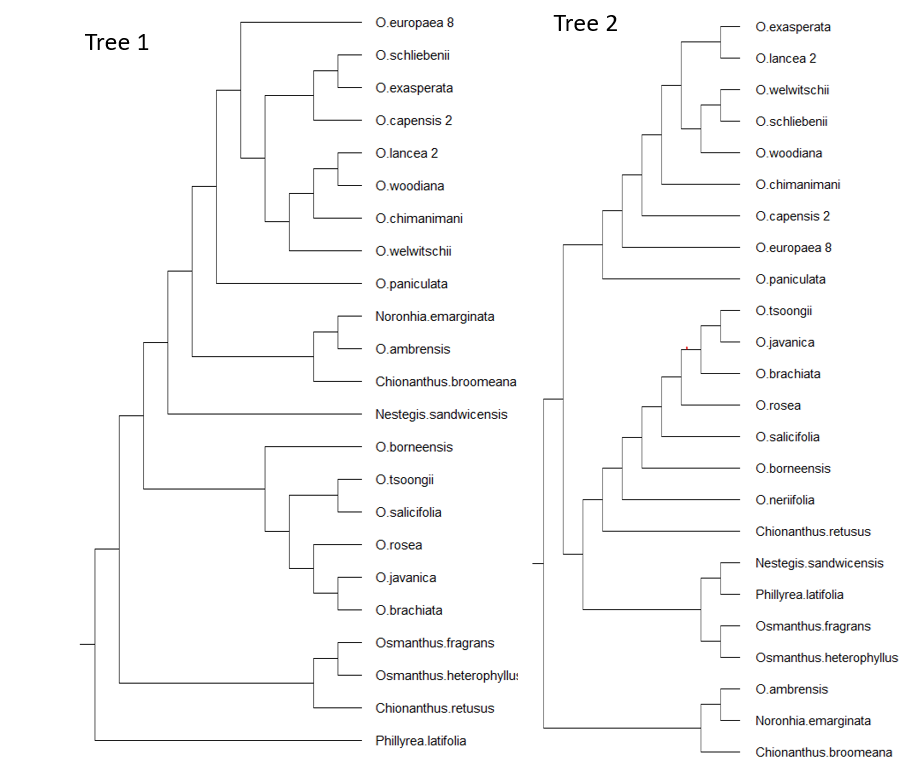


*Figure 5s*: Topologies of two trees used in this study
